# 1,5-Bis(4-iso­propyl­benzyl­idene)thio­carbonohydrazide

**DOI:** 10.1107/S1600536813027293

**Published:** 2013-10-19

**Authors:** Yan-Hua Han, Qiao Zhao, Yong Wang

**Affiliations:** a Liaocheng Technician College, Shandong 252059, People’s Republic of China; b College of Chemistry and Chemical Engineering, Liaocheng University, Shandong 252059, People’s Republic of China

## Abstract

The title compound, C_21_H_26_N_4_S, was synthesized by the condensation reaction of 4-iso­propyl­benzaldehyde with thio­carbohydrazide in ethanol. The planes of the two benzene rings in the mol­ecule are inclined at 22.6 (1)°. In the crystal, pairs of inter­molecular N—H⋯S hydrogen bonds link the mol­ecules into inversion dimers.

## Related literature

For applications of thio­carbonohydrazide derivatives, see: Bacchi *et al.* (2005 ▶); Han *et al.* (2007 ▶). For the crystal structures of related compounds, see: Gao (2013 ▶); Yu *et al.* (2013 ▶).
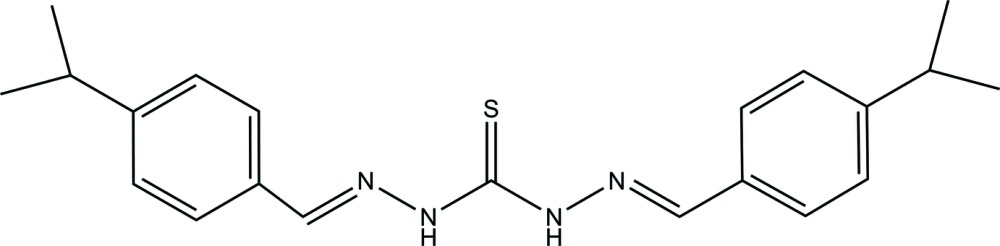


## Experimental

### 

#### Crystal data

C_21_H_26_N_4_S
*M* *_r_* = 366.52
Monoclinic, 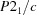

*a* = 18.082 (6) Å
*b* = 11.129 (4) Å
*c* = 10.617 (3) Å
β = 95.330 (6)°
*V* = 2127.2 (12) Å^3^
*Z* = 4
Mo *K*α radiation
μ = 0.16 mm^−1^
*T* = 296 K
0.21 × 0.18 × 0.15 mm

#### Data collection

Bruker SMART APEX diffrac­tom­eter with a CCD area detector
Absorption correction: multi-scan (*SADABS*; Bruker, 2007 ▶) *T*_min_ = 0.967, *T*_max_ = 0.976
10028 measured reflections
3659 independent reflections
1635 reflections with *I* > 2σ(*I*)
*R*_int_ = 0.065

#### Refinement

*R*[*F* ^2^ > 2σ(*F* ^2^)] = 0.078
*wR*(*F* ^2^) = 0.236
*S* = 1.08
3659 reflections
239 parameters
410 restraints
H-atom parameters constrained
Δρ_max_ = 0.38 e Å^−3^
Δρ_min_ = −0.47 e Å^−3^

### 

Data collection: *SMART* (Bruker, 2007 ▶); cell refinement: *SAINT* (Bruker, 2007 ▶); data reduction: *SAINT*; program(s) used to solve structure: *SHELXS97* (Sheldrick, 2008 ▶); program(s) used to refine structure: *SHELXL97* (Sheldrick, 2008 ▶); molecular graphics: *SHELXTL* (Sheldrick, 2008 ▶); software used to prepare material for publication: *SHELXTL*.

## Supplementary Material

Crystal structure: contains datablock(s) I, global. DOI: 10.1107/S1600536813027293/cv5428sup1.cif

Structure factors: contains datablock(s) I. DOI: 10.1107/S1600536813027293/cv5428Isup2.hkl

Supplementary material file. DOI: 10.1107/S1600536813027293/cv5428Isup3.cml

Additional supplementary materials:  crystallographic information; 3D view; checkCIF report

## Figures and Tables

**Table 1:** Hydrogen-bond geometry (Å, °)

*D*—H⋯*A*	*D*—H	H⋯*A*	*D*⋯*A*	*D*—H⋯*A*
N3—H3⋯S1^i^	0.86	2.58	3.381 (4)	155

Symmetry code: (i) 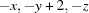.

**Fig. 1.**
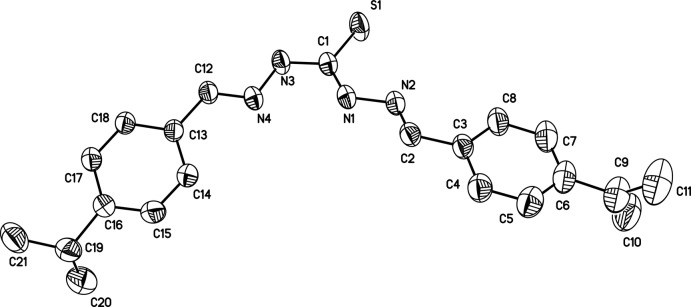
The molecular structure of (I) showing the atomic numbering and 30% probability displacement ellipsoids. H atoms omitted for clarity.

## supplementary crystallographic information

### 1. Comment

Schiff base ligands of thiocarbohydrazide have many applications in chemistry (Bacchi *et al.*, 2005; Han *et al.*, 2007). In a continuation of our structural study of thiocarbonohydrazides (Gao, 2013; Yu *et al.*, 2013), we present here the title compound (I).

In (I) (Fig. 1), the bond lengths and angles are normal and correspond to those observed in 1,5-bis(2-methoxyphenyl)methylene-thiocarbonohydrazide methanol solvate (Yu *et al.*, 2013) and 1,5-bis(1-(4-bromophenyl)ethylidene)thiocarbonohydrazide (Gao, 2013). The benzene rings C3—C8 and C13—C18 are inclined each to other at 22.6 (1)°.

In the crystal, pairs of intermolecular N—H···S hydrogen bonds (Table 1) link the molecules into centrosymmetric dimers.

### 2. Experimental

A 50 ml flask was charged with a magnetic stir bar, *p*-isopropylbenzaldehyde (2 mmol), thiocarbohydrazide (1 mmol) in 20 ml ethanol. After 5 h stirring at 373 K, the resulting mixture was cooled down to room temperature, and recrystalized from ethanol, and afforded the title compound as a crystalline solid.

### 3. Refinement

All H atoms were placed in geometrically idealized positions (C—H 0.93–0.96 Å, N—H 0.86 Å) and treated as riding on their parent atoms, with *U*_iso_(H) = 1.2–1.5*U*_eq_(C, N).

### Crystal data

**Table:**

C_21_H_26_N_4_S	*F*(000) = 784
*M**_r_* = 366.52	*D*_x_ = 1.144 Mg m^−^^3^
Monoclinic, *P*2_1_/*c*	Mo *K*α radiation, λ = 0.71073 Å
Hall symbol: -P 2ybc	Cell parameters from 1030 reflections
*a* = 18.082 (6) Å	θ = 2.7–20.2°
*b* = 11.129 (4) Å	µ = 0.16 mm^−^^1^
*c* = 10.617 (3) Å	*T* = 296 K
β = 95.330 (6)°	Block, yellow
*V* = 2127.2 (12) Å^3^	0.21 × 0.18 × 0.15 mm
*Z* = 4	

### Data collection

**Table:**

Bruker SMART APEX with a CCD area detector diffractometer	3659 independent reflections
Radiation source: fine-focus sealed tube	1635 reflections with *I* > 2σ(*I*)
Graphite monochromator	*R*_int_ = 0.065
phi and ω scans	θ_max_ = 25.0°, θ_min_ = 2.8°
Absorption correction: multi-scan (*SADABS*; Bruker, 2007)	*h* = −17→21
*T*_min_ = 0.967, *T*_max_ = 0.976	*k* = −8→13
10028 measured reflections	*l* = −12→11

### Refinement

**Table:**

Refinement on *F*^2^	Primary atom site location: structure-invariant direct methods
Least-squares matrix: full	Secondary atom site location: difference Fourier map
*R*[*F*^2^ > 2σ(*F*^2^)] = 0.078	Hydrogen site location: inferred from neighbouring sites
*wR*(*F*^2^) = 0.236	H-atom parameters constrained
*S* = 1.08	*w* = 1/[σ^2^(*F*_o_^2^) + (0.1*P*)^2^ + 0.1124*P*] where *P* = (*F*_o_^2^ + 2*F*_c_^2^)/3
3659 reflections	(Δ/σ)_max_ < 0.001
239 parameters	Δρ_max_ = 0.38 e Å^−^^3^
410 restraints	Δρ_min_ = −0.47 e Å^−^^3^

### Special details

**Table:**

Geometry. All e.s.d.'s (except the e.s.d. in the dihedral angle between two l.s. planes) are estimated using the full covariance matrix. The cell e.s.d.'s are taken into account individually in the estimation of e.s.d.'s in distances, angles and torsion angles; correlations between e.s.d.'s in cell parameters are only used when they are defined by crystal symmetry. An approximate (isotropic) treatment of cell e.s.d.'s is used for estimating e.s.d.'s involving l.s. planes.
Refinement. Refinement of *F*^2^ against ALL reflections. The weighted *R*-factor *wR* and goodness of fit *S* are based on *F*^2^, conventional *R*-factors *R* are based on *F*, with *F* set to zero for negative *F*^2^. The threshold expression of *F*^2^ > σ(*F*^2^) is used only for calculating *R*-factors(gt) *etc*. and is not relevant to the choice of reflections for refinement. *R*-factors based on *F*^2^ are statistically about twice as large as those based on *F*, and *R*- factors based on ALL data will be even larger.

### Fractional atomic coordinates and isotropic or equivalent isotropic displacement parameters (Å^2^)

**Table:**

	*x*	*y*	*z*	*U*_iso_*/*U*_eq_	
N1	0.1303 (2)	0.8272 (4)	0.2266 (4)	0.0648 (11)	
H1	0.1163	0.8211	0.3016	0.078*	
N2	0.1942 (2)	0.7702 (4)	0.1996 (4)	0.0649 (11)	
N3	0.0291 (2)	0.9425 (4)	0.1818 (3)	0.0680 (11)	
H3	0.0049	0.9959	0.1361	0.082*	
N4	0.0053 (2)	0.9097 (3)	0.2969 (3)	0.0621 (10)	
S1	0.11024 (8)	0.91415 (15)	−0.00810 (13)	0.0919 (6)	
C1	0.0896 (3)	0.8915 (5)	0.1412 (4)	0.0630 (12)	
C2	0.2323 (3)	0.7216 (4)	0.2937 (5)	0.0690 (12)	
H2	0.2172	0.7302	0.3746	0.083*	
C3	0.2989 (3)	0.6530 (5)	0.2757 (5)	0.0693 (12)	
C4	0.3340 (3)	0.5859 (5)	0.3754 (6)	0.0888 (15)	
H4	0.3173	0.5904	0.4555	0.107*	
C5	0.3926 (3)	0.5138 (6)	0.3552 (7)	0.1026 (16)	
H5	0.4145	0.4697	0.4231	0.123*	
C6	0.4212 (3)	0.5025 (6)	0.2414 (7)	0.1014 (16)	
C7	0.3874 (3)	0.5705 (6)	0.1433 (7)	0.1014 (15)	
H7	0.4053	0.5660	0.0641	0.122*	
C8	0.3284 (3)	0.6442 (5)	0.1592 (6)	0.0869 (14)	
H8	0.3075	0.6893	0.0912	0.104*	
C9	0.4818 (4)	0.4181 (7)	0.2203 (8)	0.1266 (19)	
H9	0.4762	0.3718	0.2974	0.152*	
C10	0.4599 (4)	0.3124 (7)	0.1311 (9)	0.156 (3)	
H10A	0.4395	0.3429	0.0507	0.234*	
H10B	0.4235	0.2636	0.1673	0.234*	
H10C	0.5031	0.2648	0.1197	0.234*	
C11	0.5593 (4)	0.4607 (8)	0.2642 (10)	0.176 (3)	
H11A	0.5871	0.4714	0.1923	0.265*	
H11B	0.5835	0.4020	0.3200	0.265*	
H11C	0.5565	0.5357	0.3081	0.265*	
C12	−0.0521 (3)	0.9657 (5)	0.3280 (4)	0.0636 (12)	
H12	−0.0709	1.0296	0.2783	0.076*	
C13	−0.0890 (2)	0.9318 (4)	0.4397 (4)	0.0607 (11)	
C14	−0.0643 (3)	0.8389 (5)	0.5184 (5)	0.0752 (13)	
H14	−0.0213	0.7975	0.5034	0.090*	
C15	−0.1028 (3)	0.8071 (5)	0.6185 (5)	0.0846 (14)	
H15	−0.0838	0.7461	0.6720	0.102*	
C16	−0.1689 (3)	0.8621 (5)	0.6435 (5)	0.0716 (12)	
C17	−0.1916 (3)	0.9581 (5)	0.5653 (5)	0.0731 (12)	
H17	−0.2343	1.0001	0.5804	0.088*	
C18	−0.1526 (3)	0.9926 (5)	0.4665 (4)	0.0699 (12)	
H18	−0.1691	1.0577	0.4166	0.084*	
C19	−0.2112 (3)	0.8149 (5)	0.7515 (6)	0.0901 (16)	
H19	−0.1737	0.7960	0.8213	0.108*	
C20	−0.2469 (4)	0.6994 (6)	0.7122 (7)	0.125 (2)	
H20A	−0.2697	0.6653	0.7820	0.188*	
H20B	−0.2100	0.6450	0.6860	0.188*	
H20C	−0.2840	0.7131	0.6430	0.188*	
C21	−0.2629 (4)	0.9014 (7)	0.8022 (6)	0.119 (2)	
H21A	−0.3039	0.9159	0.7400	0.178*	
H21B	−0.2373	0.9755	0.8224	0.178*	
H21C	−0.2810	0.8689	0.8773	0.178*	

### Atomic displacement parameters (Å^2^)

**Table:**

	*U*^11^	*U*^22^	*U*^33^	*U*^12^	*U*^13^	*U*^23^
N1	0.058 (2)	0.076 (3)	0.063 (2)	0.002 (2)	0.0203 (19)	−0.008 (2)
N2	0.056 (2)	0.071 (3)	0.070 (3)	−0.001 (2)	0.017 (2)	−0.010 (2)
N3	0.064 (2)	0.085 (3)	0.058 (2)	0.010 (2)	0.0209 (19)	−0.006 (2)
N4	0.058 (2)	0.074 (3)	0.056 (2)	−0.002 (2)	0.0155 (18)	−0.0111 (19)
S1	0.0794 (10)	0.1351 (15)	0.0648 (8)	0.0252 (9)	0.0251 (7)	0.0002 (8)
C1	0.058 (3)	0.073 (3)	0.060 (3)	0.002 (2)	0.016 (2)	−0.009 (2)
C2	0.060 (2)	0.068 (3)	0.081 (3)	−0.006 (2)	0.019 (2)	−0.007 (2)
C3	0.056 (2)	0.067 (3)	0.087 (3)	−0.005 (2)	0.016 (2)	−0.009 (2)
C4	0.073 (3)	0.092 (3)	0.103 (3)	0.002 (3)	0.013 (3)	−0.005 (3)
C5	0.083 (3)	0.098 (3)	0.125 (3)	0.010 (3)	0.001 (3)	−0.008 (3)
C6	0.071 (3)	0.100 (3)	0.133 (4)	0.013 (3)	0.012 (3)	−0.021 (3)
C7	0.074 (3)	0.109 (3)	0.124 (3)	0.007 (3)	0.024 (3)	−0.019 (3)
C8	0.063 (3)	0.094 (3)	0.106 (3)	0.004 (2)	0.022 (2)	−0.008 (3)
C9	0.094 (4)	0.124 (4)	0.161 (4)	0.018 (3)	0.010 (3)	−0.023 (4)
C10	0.127 (5)	0.125 (5)	0.218 (7)	0.023 (5)	0.018 (5)	−0.034 (5)
C11	0.118 (5)	0.178 (7)	0.231 (8)	0.042 (5)	0.001 (5)	−0.047 (6)
C12	0.061 (2)	0.072 (3)	0.060 (2)	0.009 (2)	0.015 (2)	0.000 (2)
C13	0.060 (2)	0.064 (3)	0.059 (2)	0.009 (2)	0.011 (2)	−0.001 (2)
C14	0.068 (3)	0.076 (3)	0.084 (3)	0.014 (2)	0.019 (2)	0.011 (2)
C15	0.083 (3)	0.081 (3)	0.092 (3)	0.010 (2)	0.014 (2)	0.022 (2)
C16	0.070 (3)	0.071 (3)	0.075 (3)	0.002 (2)	0.018 (2)	0.005 (2)
C17	0.067 (2)	0.080 (3)	0.075 (3)	0.014 (2)	0.019 (2)	0.004 (2)
C18	0.072 (3)	0.074 (3)	0.066 (2)	0.019 (2)	0.016 (2)	0.009 (2)
C19	0.086 (3)	0.086 (4)	0.101 (3)	−0.005 (3)	0.022 (3)	0.016 (3)
C20	0.126 (5)	0.117 (5)	0.136 (5)	−0.017 (4)	0.033 (4)	0.013 (4)
C21	0.119 (5)	0.128 (5)	0.117 (5)	−0.020 (4)	0.051 (4)	−0.003 (4)

### Geometric parameters (Å, º)

**Table:**

N1—C1	1.324 (6)	C10—H10C	0.9600
N1—N2	1.370 (5)	C11—H11A	0.9600
N1—H1	0.8600	C11—H11B	0.9600
N2—C2	1.279 (6)	C11—H11C	0.9600
N3—C1	1.339 (5)	C12—C13	1.463 (6)
N3—N4	1.381 (5)	C12—H12	0.9300
N3—H3	0.8600	C13—C14	1.376 (6)
N4—C12	1.280 (5)	C13—C18	1.387 (6)
S1—C1	1.681 (5)	C14—C15	1.369 (7)
C2—C3	1.453 (6)	C14—H14	0.9300
C2—H2	0.9300	C15—C16	1.390 (7)
C3—C8	1.394 (7)	C15—H15	0.9300
C3—C4	1.399 (7)	C16—C17	1.392 (7)
C4—C5	1.362 (8)	C16—C19	1.530 (7)
C4—H4	0.9300	C17—C18	1.371 (6)
C5—C6	1.363 (8)	C17—H17	0.9300
C5—H5	0.9300	C18—H18	0.9300
C6—C7	1.383 (9)	C19—C21	1.477 (8)
C6—C9	1.477 (9)	C19—C20	1.480 (8)
C7—C8	1.369 (7)	C19—H19	0.9800
C7—H7	0.9300	C20—H20A	0.9600
C8—H8	0.9300	C20—H20B	0.9600
C9—C11	1.511 (8)	C20—H20C	0.9600
C9—C10	1.538 (8)	C21—H21A	0.9600
C9—H9	0.9800	C21—H21B	0.9600
C10—H10A	0.9600	C21—H21C	0.9600
C10—H10B	0.9600		
			
C1—N1—N2	122.2 (4)	C9—C11—H11B	109.5
C1—N1—H1	118.9	H11A—C11—H11B	109.5
N2—N1—H1	118.9	C9—C11—H11C	109.5
C2—N2—N1	115.9 (4)	H11A—C11—H11C	109.5
C1—N3—N4	120.3 (4)	H11B—C11—H11C	109.5
C1—N3—H3	119.9	N4—C12—C13	121.7 (4)
N4—N3—H3	119.9	N4—C12—H12	119.2
C12—N4—N3	115.3 (4)	C13—C12—H12	119.2
N1—C1—N3	115.3 (4)	C14—C13—C18	118.0 (4)
N1—C1—S1	124.7 (3)	C14—C13—C12	122.7 (4)
N3—C1—S1	120.0 (4)	C18—C13—C12	119.3 (4)
N2—C2—C3	120.8 (5)	C15—C14—C13	120.4 (5)
N2—C2—H2	119.6	C15—C14—H14	119.8
C3—C2—H2	119.6	C13—C14—H14	119.8
C8—C3—C4	116.7 (5)	C14—C15—C16	122.8 (5)
C8—C3—C2	122.9 (5)	C14—C15—H15	118.6
C4—C3—C2	120.3 (5)	C16—C15—H15	118.6
C5—C4—C3	120.0 (6)	C15—C16—C17	115.8 (4)
C5—C4—H4	120.0	C15—C16—C19	119.4 (5)
C3—C4—H4	120.0	C17—C16—C19	124.8 (5)
C4—C5—C6	123.9 (7)	C18—C17—C16	121.8 (5)
C4—C5—H5	118.0	C18—C17—H17	119.1
C6—C5—H5	118.0	C16—C17—H17	119.1
C5—C6—C7	116.1 (6)	C17—C18—C13	121.0 (5)
C5—C6—C9	122.7 (7)	C17—C18—H18	119.5
C7—C6—C9	121.1 (7)	C13—C18—H18	119.5
C8—C7—C6	122.0 (6)	C21—C19—C20	113.3 (5)
C8—C7—H7	119.0	C21—C19—C16	115.3 (5)
C6—C7—H7	119.0	C20—C19—C16	108.8 (5)
C7—C8—C3	121.2 (6)	C21—C19—H19	106.3
C7—C8—H8	119.4	C20—C19—H19	106.3
C3—C8—H8	119.4	C16—C19—H19	106.3
C6—C9—C11	115.7 (6)	C19—C20—H20A	109.5
C6—C9—C10	115.3 (6)	C19—C20—H20B	109.5
C11—C9—C10	127.4 (7)	H20A—C20—H20B	109.5
C6—C9—H9	94.1	C19—C20—H20C	109.5
C11—C9—H9	94.1	H20A—C20—H20C	109.5
C10—C9—H9	94.1	H20B—C20—H20C	109.5
C9—C10—H10A	109.5	C19—C21—H21A	109.5
C9—C10—H10B	109.5	C19—C21—H21B	109.5
H10A—C10—H10B	109.5	H21A—C21—H21B	109.5
C9—C10—H10C	109.5	C19—C21—H21C	109.5
H10A—C10—H10C	109.5	H21A—C21—H21C	109.5
H10B—C10—H10C	109.5	H21B—C21—H21C	109.5
C9—C11—H11A	109.5		

### Hydrogen-bond geometry (Å, º)

**Table:**

*D*—H···*A*	*D*—H	H···*A*	*D*···*A*	*D*—H···*A*
N3—H3···S1^i^	0.86	2.58	3.381 (4)	155

Symmetry code: (i) −*x*, −*y*+2, −*z*.
